# The Cumming's Patent

**Published:** 1872-05

**Authors:** 


*The Cumming's Patent.*—As we go to press we hear that the Supreme Court of the U. S., has decided in favor of the validity of the "Cumming's Patent."

